# Solving Gravity Anomaly Matching Problem Under Large Initial Errors in Gravity Aided Navigation by Using an Affine Transformation Based Artificial Bee Colony Algorithm

**DOI:** 10.3389/fnbot.2019.00019

**Published:** 2019-05-08

**Authors:** Tian Dai, Lingjuan Miao, Haijun Shao, Yongsheng Shi

**Affiliations:** School of Automation, Beijing Institute of Technology, Beijing, China

**Keywords:** gravity aided navigation, bio-inspired navigation, navigation systems, optimization, underwater vehicle, evolutionary algorithm

## Abstract

Gravity aided inertial navigation system (GAINS), which uses earth gravitational anomaly field for navigation, holds strong potential as an underwater navigation system. The gravity matching algorithm is one of the key factors in GAINS. Existing matching algorithms cannot guarantee the matching accuracy in the matching algorithms based gravity aided navigation when the initial errors are large. Evolutionary algorithms, which are mostly have the ability of global optimality and fast convergence, can be used to solve the gravity matching problem under large initial errors. However, simply applying evolutionary algorithms to GAINS may lead to false matching. Therefore, in order to deal with the underwater gravity matching problem, it is necessary to improve the traditional evolutionary algorithms. In this paper, an affine transformation based artificial bee colony (ABC) algorithm, which can greatly improve the positioning precision under large initial errors condition, is developed. The proposed algorithm introduces affine transformation to both initialization process and evolutionary process of ABC algorithm. The single-point matching strategy is replaced by the strategy of matching a sequence of several consecutive position vectors. In addition, several constraints are introduced to the process of evolution by using the output characteristics of the inertial navigation system (INS). Simulations based on the actual gravity anomaly base map have been performed for the validation of the proposed algorithm.

## Introduction

It is well known that inertial navigation systems (INSs) typically used on underwater vehicles tend to develop accumulated errors. Above water, the INS data can be corrected by the use of global navigation satellite system (GNSS) (Bishop, 2002). However, due to the rapid attenuation of higher frequency signals and the unstructured nature of the undersea environment, GNSS signals can only propagate within short distance under water (Paull et al., 2013). As a passive navigation system, the gravity aided inertial navigation system (GAINS) holds strong potential as an auxiliary navigation system (Canciani and Raquet, 2017). GAINS obtains a gravity anomaly measurements by using gravimeters installed on a vehicle. These measurements are matched with a priori digital map of the gravity anomaly, to estimate the vehicle position. GAINSs are completely passive and difficult to interfere with. These advantages are totally meet the requirement of underwater vehicles (Rice et al., 2004).

A GAINS consists of a priori gravity anomaly database (gravity anomaly map), a measurement unit (gravimeter), and a navigation algorithm. There are two types of GAINSs: filtering algorithms based GAINSs (Li et al., 2013; Claus and Bachmayer, 2015; Copp and Subbarao, 2015; Allotta et al., 2016) and matching algorithms based GAINSs (Zhao et al., 2014; Wu et al., 2015; Zhu et al., 2015; Han et al., 2016; Song et al., 2016). Filtering algorithms, which take use of simplified dynamic mathematical model of the vehicle, have a good performance in real-time. In filtering algorithms based GAINSs, commonly used filters are the extended Kalman filter (EKF), the particle filter (PF) and the Rao-Blackwellized particle filter (RBPF). EKF is more effective for low nonlinear estimation problems. PF can effectively handle highly non-linear or non-Gaussian estimation problems. RBPF is a hybrid filter combining EKF and PF (Simanek et al., 2015; Kim T. et al., 2018; Kim Y. et al., 2018). However, the application of filtering algorithms is limited since the precise model of gravity anomaly is difficult to establish (Han et al., 2016). The matching algorithm is a key factor in matching algorithms based GAINSs (Hegrenaes and Hallingstad, 2011; Wu et al., 2017). Terrain contour matching (TERCOM) (Affleck and Jircitano, 1990) algorithm and iterative closest contour point (ICCP) (Kamgarparsi and Kamgarparsi, 1999) algorithm are two conventional matching algorithms in GAINSs. TERCOM algorithm is realized via group correlation analysis. Most of the improvements to the TERCOM algorithm (Zhao et al., 2014; Han et al., 2016) are proposed to solve bad real-time performance and heavy computational complexity problem. However, TERCOM algorithm is sensitive to angular error of the INS-indicated segment, which is difficult to improve. ICCP algorithm, which uses rigid transformation to matching the multilateral arc, has a high matching accuracy. Over the past decade, a number of improved ICCP algorithm have been suggested. Among them, a part of researches were developed for improving the real-time performance (Tong et al., 2011; Wang et al., 2013). Other studies adopted affine transformation to deal with the scale error (Xu et al., 2014; Song et al., 2016). However, the mismatching of ICCP algorithm is easily included when the initial position errors of INS are large (Han et al., 2016). Both TERCOM-based algorithms and ICCP-based algorithms cannot guarantee the matching accuracy when initial errors are large. TERCOM-based algorithms are sensitive to angular errors of the INS-indicated segments and ICCP-based algorithms are sensitive to position errors. Therefore, a matching algorithm that maintain high matching precision under large initial errors is needed. Evolutionary algorithms (Quan and Fang, 2010; Gao et al., 2014; Hidalgo-Paniagua et al., 2016; Teymourian et al., 2016; Li et al., 2017) are a good choice because most of them have the ability of both global optimality and fast convergence.

The evolutionary algorithms have emerged as a powerful tool for finding optimum solutions of complex optimization problems. In the past few decades, a number of evolutionary algorithms have been used extensively to obtain optimal designs and overcome the computational drawbacks of traditional mathematical optimization methods (Abrao, 2013; Yildiz, 2013). Jaesung and Kim improved the genetic algorithm so solve robot path planning problem (Lee and Kim, 2016). An improved intelligent water drops algorithm and an advanced cuckoo search algorithm are proposed to solve the capacitated vehicle routing problem (Teymourian et al., 2016). Improved bee colony algorithm is applied to the color image segmentation, assigning the optimal coordinates of seeds and determining similarity differences (Sag and Çunkaş, 2015).

Gravity matching problem can be regarded as an optimization problem (Wu et al., 2017). Therefore, evolutionary algorithms can be directly applied to the gravity matching problem. Gao et al. proposed an improved artificial bee colony (ABC) algorithm and obtained a good performance on gravity matching under small initial errors (Gao et al., 2014). However, there exists a lot of similarity feature points in matching area when initial errors are large. Although the improved ABC algorithm can be implemented on navigation systems under large initial errors, the mismatching of position is easily included. In this paper, we consider a challenging gravity matching problem of achieving high precision under large initial errors. To solve this problem, we propose an affine transformation based ABC algorithm. Firstly, In order to avoid mismatching caused by similarity feature points, we apply sequence matching strategy to the proposed algorithm. Secondly, Scaling transformation, rotation transformation, and translation transformation are introduced to both initialization process and evolutionary process to increase the convergence speed. In addition, we constrain the affine transformation by utilizing the output characteristics of INS.

The rest of the paper is organized as follows. The second part introduces the ABC algorithm. INS error propagation model and the constraints of affine transformation are provided in the third part. The fourth part of this paper presents the procedure of affine transformation based artificial bee colony algorithm. A comprehensive discussion on the experimental settings and simulation results are provided in the fifth part. Conclusions are given in the last part.

## Artificial Bee Colony Algorithm

The ABC algorithm (Karaboga and Basturk, 2007) is one of the most recently introduced swarm-based search methods (Akay and Karaboga, 2012; Akbari et al., 2012; Sag and Çunkaş, 2015; Li et al., 2016). It contains three groups of bees: employed bees, onlookers, and scouts. In ABC algorithm, the position of a food source represents a possible solution of the optimization problem and the nectar amount of a food source corresponds to the quality (fitness) of the associated solution. The more a solution has high fitness, the more possibility of being selected of this solution by onlooker bees. The number of the employed bees or the onlooker bees is equal to the number of solutions in the population. The initial swarm composed of employed bees are generated by (1):

(1) $$\begin{array}{r} {\text{x}}_{{}_{i}}^{j}={\text{x}}_{\min}^{j}+rand\left(0,1\right)\left({\text{x}}_{\max}^{j}-{\text{x}}_{\min}^{j}\right) \end{array}$$

Where ${\text{x}}_{{}_{i}}^{j}$ indicates the *j*th parameter of *i*th solution in the population, *i* = {1, 2, ⋯ , *SN*} and *SN* is the size of population, *j* = {1, 2, ⋯ , *D*} and *D* is the number of parameters in a solution. ${\text{x}}_{\min}^{j}$ and ${\text{x}}_{\max}^{j}$ are, respectively, the lower bound and the upper bound of *j*th parameter. *rand*(0, 1) generates a real number between 0 and 1.

After initialization, fitness values of solutions are calculated by (2):

(2) $$fit_{i}=\{\begin{array}{cc} 1/(1+f_{i}) & f_{i}\geq 0\\ 1+\left|f_{i}\right| & f_{i}<0 \end{array}$$

Where *f*_*i*_ is the objective function value of *i*th solution in the population. *fit*_*i*_ is the fitness value of *i*th solution in the population. The employed bee evaluates the quality of food sources and determines a new one according to the fitness value. If the nectar amount of new source is better than the old source, employed bee will update its memory with new source. The employed bees update their sources according to the following equation:

(3) $$\begin{array}{r} {\text{v}}_{{}_{i}}^{j}={\text{x}}_{{}_{i}}^{j}+rand\left(-1,1\right)\left({\text{x}}_{{}_{i}}^{j}-{\text{x}}_{neighbor}^{j}\right) \end{array}$$

Where ${\text{x}}_{neighbor}^{j}$ is the neighbor solution selected randomly, neighbor ∈ {1, 2, ⋯ , *i* − 1, *i* + 1, ⋯ , *SN*}. ${\text{v}}_{{}_{i}}^{j}$ represents new solution.

After new food sources have been explored, onlookers select an employed bee for guidance. For this purpose, roulette wheel is used to calculate the probabilities:

(4) $$\begin{array}{r} p_{i}=\frac{fit_{i}}{\overset{SN}{\underset{q=1}{\sum }}fit_{q}} \end{array}$$

After the onlooker bees select a food source as a guide, the candidate food source is calculated by (3). Afterwards, the greedy selection process is applied for the onlooker bees. The best solution achieved so far is memorized. In addition, a food source will be abandoned when limit, a control parameter, is exceeded for the source. So it is replaced with randomly produced solution by (1).

For the gravity matching problem, denote ***x*** as the position of vehicle and *f* as the gravity objective function. Then ABC algorithm can be directly used to find the optimal position. However, simply applying ABC algorithm to the gravity matching problem may lead to false matching (Gao et al., 2014). There exists a lot of similarity gravity feature points in gravity database, which means single-point matching process may easily convergence at the wrong position.

## Sequence Matching Strategy

To solve the mismatching problem, the single-point matching strategy in basic ABC is replaced by strategy of matching a sequence of several consecutive position vectors. Considering the short-term high-precision characteristics of the INS (Wu et al., 2015), affine transformation could be used to describe the relationship between the sequence to be matched and the INS-indicated sequence. Specifically, these transformations include scaling transformation, rotation transformation, and translation transformation. The range of affine transformations can be estimated by an INS error propagation model.

### INS Error Propagation Model

Affine transformation is constrained by the output characteristics of the INS. Therefore, an INS error propagation model (Yan et al., 2008) is employed in this paper. In Yan et al. (2008), i-reference frame is the fixed inertial frame; e-reference frame is the Terrestrial reference frame (TRF); n-reference frame has its origin on the surface and its axes pointing East, North, and Up (ENU reference frame); b-reference frame is centered in the center of gravity of the vehicle, with the *y*-axis pointing in the direction of the forward motion of the vehicle, the *z*-axis pointing up and the *x*-axis completing a right-handed reference frame. However, GAINSs are commonly used in underwater environments. In which case n-reference frame adopts North, East and Down (NED) reference frame; b-reference frame is centered in the center of gravity of the vehicle, with the *x*-axis pointing in the direction of the forward motion of the vehicle, the *z*-axis pointing down and the *y*-axis completing a right-handed reference frame (Allotta et al., 2016). Hence the INS error propagation model in Yan et al. (2008) needs to be modified.

In actual case, there exist rotation errors between n-reference frame (ideal mathematics platform) and n’-reference frame (actual mathematics platform). n’-reference frame can be gained after rotating 3 times of n-reference frame. Let α_*z*_, α_*y*_, α_*x*_ be the three rotational angles and ***α*** = [*α*_*x*_ *α*_*y*_ *α*_*z*_], the rotation matrixes can be expressed as:

(5) $$\begin{array}{r} \begin{array}{cc} C_{\alpha_{z}} & =\left[\begin{array}{ccc} \cos\alpha_{z} & -\sin\alpha_{z} & 0\\ \sin\alpha_{z} & \cos\alpha_{z} & 0\\ 0 & 0 & 1 \end{array}\right]\\ C_{\alpha_{y}} & =\left[\begin{array}{ccc} \cos\alpha_{y} & 0 & -\sin\alpha_{y}\\ 0 & 1 & 0\\ \sin\alpha_{y} & 0 & \cos\alpha_{y} \end{array}\right]\\ C_{\alpha_{x}} & =\left[\begin{array}{ccc} 1 & 0 & 0\\ 0 & \cos\alpha_{x} & \sin\alpha_{x}\\ 0 & -\sin\alpha_{x} & \cos\alpha_{x} \end{array}\right] \end{array} \end{array}$$

Then, the coordinate transformation matrix from n-reference frame to n’-reference frame is derived:

(6) $$\begin{array}{r} {\text{C}}_{\text{n}}^{{\text{n}}^{\prime }\;}={\text{C}}_{\alpha_{x}}{\text{C}}_{\alpha_{y}}{\text{C}}_{\alpha_{z}} \end{array}$$

Denoting with $\omega_{{\text{nn}}^{\prime }\;}^{{\text{n}}^{\prime }\;}$ the relative angular velocity of n′-reference frame in n-reference frame, the following equation has been derived:

(7) $$\begin{array}{r} \omega_{{\text{nn}}^{\prime }\;}^{{\text{n}}^{\prime }\;}={\text{C}}_{\alpha_{x}}{\text{C}}_{\alpha_{y}}\left[\begin{array}{c} 0\\ 0\\ {\dot{\alpha }}_{z} \end{array}\right]+{\text{C}}_{\alpha_{x}}\left[\begin{array}{c} 0\\ {\dot{\alpha }}_{y}\\ 0 \end{array}\right]+\left[\begin{array}{c} {\dot{\alpha }}_{x}\\ 0\\ 0 \end{array}\right]={\text{C}}_{\omega }\left[\begin{array}{c} {\dot{\alpha }}_{x}\\ {\dot{\alpha }}_{y}\\ {\dot{\alpha }}_{z} \end{array}\right] \end{array}$$

Hence, the differential equation of Euler platform error angles can be expressed as follows:

(8) $$\dot{\alpha }={\text{C}}_{\omega }^{-1}\omega_{{\text{nn}}^{\text{′}}}^{{\text{n}}^{\text{′}}}$$

Here **C**_ω_ and ${\text{C}}_{\omega }^{\text{-}1}$ are calculated by the following expressions:

(9) $$\begin{array}{r} \begin{array}{c} {\text{C}}_{\omega }=\left[\begin{array}{ccc} 1 & 0 & -\sin\alpha_{y}\\ 0 & \cos\alpha_{x} & \sin\alpha_{x}\cos\alpha_{y}\\ 0 & -\sin\alpha_{x} & \cos\alpha_{x}\cos\alpha_{y} \end{array}\right]\\ {\text{C}}_{\omega }^{-1}=\frac{1}{\cos\alpha_{y}}\left[\begin{array}{ccc} \cos\alpha_{y} & \sin\alpha_{x}\sin\alpha_{y} & \cos\alpha_{x}\sin\alpha_{y}\\ 0 & \cos\alpha_{x}\cos\alpha_{y} & -\sin\alpha_{x}\cos\alpha_{y}\\ 0 & \sin\alpha_{x} & \cos\alpha_{x} \end{array}\right] \end{array} \end{array}$$

Denoting with ${\text{ω}}_{\text{ie}}^{\text{n}}$ the rotational angular velocity of the earth. *L* and *h* are, respectively, latitude, and depth. *R*_M_ is the local radius of curvature in meridian and *R*_N_ is the local radius of curvature in prime vertical. $\hat{L}=L+\delta L$, ĥ = *h* + δ*h*. In addition, δ*L* and δ*h* are slight errors. The following equations have been derived:

(10) $$\begin{array}{rcl} \omega_{\text{ie}}^{\text{n}} & = & {\left[\begin{array}{ccc} \omega_{\text{ie}}\cos L & 0 & -\omega_{\text{ie}}\sin L \end{array}\right]}^{\text{T}} \end{array}$$

(11) $$\begin{array}{rcl} \omega_{\text{en}}^{\text{n}} & = & {\left[\begin{array}{ccc} \frac{v_{\text{E}}^{\text{n}}}{R_{\text{N}}-h} & -\frac{v_{\text{N}}^{\text{n}}}{R_{\text{M}}-h} & -\frac{v_{\text{E}}^{\text{n}}}{R_{\text{N}}-h}\tan L \end{array}\right]}^{\text{T}} \end{array}$$

(12) $$\begin{array}{rcl} \delta {\text{ω}}_{\text{ie}}^{\text{n}} & = & \left[\begin{array}{c} -\omega_{\text{ie}}\sin\hat{L}\delta L\\ 0\\ -\omega_{\text{ie}}\cos\hat{L}\delta L \end{array}\right] \end{array}$$

(13) $$\begin{array}{rcl} \delta {\text{ω}}_{\text{en}}^{\text{n}} & = & \left[\begin{array}{c} \delta v_{\text{E}}^{\text{n}}/\left({\hat{R}}_{\text{N}}-ĥ\right)\\ -\delta v_{\text{N}}^{\text{n}}/\left({\hat{R}}_{\text{M}}-ĥ\right)\\ -\left(\tan\hat{L}\delta v_{\text{E}}^{\text{n}}+{\hat{v}}_{\text{E}}^{\text{n}}\sec^{2}\hat{L}\delta L\right)/\left({\hat{R}}_{\text{N}}-ĥ\right) \end{array}\right] \end{array}$$

${\text{ω}}_{\text{in}}^{\text{n}}$ and $\delta {\text{ω}}_{\text{in}}^{\text{n}}$ can be expressed as follows:

(14) $$\begin{array}{rcl} \omega_{\text{in}}^{\text{n}} & = & {\text{ω}}_{\text{ie}}^{\text{n}}+{\text{ω}}_{\text{en}}^{\text{n}} \end{array}$$

(15) $$\begin{array}{rcl} \delta {\text{ω}}_{\text{in}}^{\text{n}} & = & \delta {\text{ω}}_{\text{ie}}^{\text{n}}+\delta {\text{ω}}_{\text{en}}^{\text{n}} \end{array}$$

On the basis of these formulas, the INS attitude error and velocity error have been derived (Yan et al., 2008):

(16) $$\dot{\alpha }={\text{C}}_{\omega }^{\text{-}1}\left[\left(I\text{-}{\text{C}}_{\text{n}}^{\text{n}\prime }\right){\hat{\omega }}_{\text{in}}^{\text{n}}+{\text{C}}_{\text{n}}^{\text{n}\prime }\delta \omega_{\text{in}}^{\text{n}}-{\text{C}}_{\text{b}}^{\text{n}\prime }\delta \omega_{\text{ib}}^{\text{b}}\right]$$

(17) $$\begin{array}{ll} \delta {\dot{\text{v}}}^{\text{n}} & =\left[\text{I}\text{-}{\left({\text{C}}_{\text{n}}^{\text{n}\prime }\right)}^{\text{T}}\right]{\text{C}}_{\text{b}}^{\text{n}\prime }{\hat{\text{f}}}_{\text{sf}}^{\text{b}}+{\left({\text{C}}_{\text{n}}^{\text{n}\prime }\right)}^{\text{T}}{\text{C}}_{\text{b}}^{\text{n}\prime }\delta {\text{f}}_{\text{sf}}^{\text{b}}-\left(2\delta \omega_{\text{ie}}^{\text{n}}+\delta \omega_{\text{en}}^{\text{n}}\right)\\ & \times \left({\hat{\text{v}}}^{\text{n}}-\delta {\text{v}}^{\text{n}}\right)-\left(2{\hat{\omega }}_{\text{ie}}^{\text{n}}+{\hat{\omega }}_{\text{en}}^{\text{n}}\right)\times \delta {\text{v}}^{\text{n}}+\delta {\text{g}}^{\text{n}} \end{array}$$

Where $\delta \omega_{\text{ib}}^{\text{b}}$ is the measurement error of gyroscope, $\delta {\text{ω}}_{\text{in}}^{\text{n}}$ is the calculation error of ${\text{ω}}_{\text{in}}^{\text{n}}$. $\delta f_{\text{sf}}^{\text{b}}$ is the measurement error of accelerometer. In addition, $\delta {\text{ω}}_{\text{ib}}^{\text{b}}$ are mainly consist of a constant bias ***ε***^b^ and zero mean Gaussian white noise ${\text{w}}_{\text{g}}^{\text{b}}$. $\delta f_{\text{sf}}^{\text{b}}$ are mainly consist of a constant bias ∇^b^ and zero mean Gaussian white noise ${\text{w}}_{\text{a}}^{\text{b}}$. Besides, δ***g***^n^could be ignored. Hence, the INS error propagation model used in underwater environment is defined by the following equations:

(18) $$\{\begin{array}{l} \delta \dot{L}\text{=}\frac{\delta v_{\text{N}}^{\text{n}}}{R_{\text{M}}-h}+\delta h\frac{v_{\text{N}}^{\text{n}}}{{\left(R_{\text{M}}-h\right)}^{2}}\\ \delta \dot{\lambda }\text{=}\frac{\delta v_{\text{E}}^{\text{n}}}{R_{\text{N}}-h}\sec L+\delta L\frac{v_{\text{E}}^{\text{n}}}{R_{\text{N}}-h}\tan L\sec L+\delta h\frac{v_{\text{E}}^{\text{n}}\sec L}{{\left(R_{\text{N}}-h\right)}^{2}}\\ \delta \dot{h}\text{=}\delta V_{\text{D}}\\ \dot{\alpha }\text{=}{\text{C}}_{\omega }^{\text{-}1}\left[\left(\text{I}\text{-}{\text{C}}_{\text{n}}^{\text{n}\prime }\right){\hat{\omega }}_{\text{in}}^{\text{n}}+{\text{C}}_{\text{n}}^{\text{n}\prime }\delta \omega_{\text{in}}^{\text{n}}-{\text{C}}_{\text{b}}^{\text{n}\prime }\varepsilon^{\text{b}}\right]-{\text{C}}_{\omega }^{\text{-}1}{\text{C}}_{\text{b}}^{\text{n}\prime }{\text{w}}_{\text{g}}^{\text{b}}\\ \delta {\dot{v}}^{\text{n}}\text{=}\left[\text{-}{\left({\text{C}}_{\text{n}}^{\text{n}\prime }\right)}^{\text{T}}\right]C_{\text{b}}^{\text{n}\prime }{\hat{f}}_{\text{sf}}^{\text{b}}+{\left({\text{C}}_{\text{n}}^{\text{n}\prime }\right)}^{\text{T}}{\text{C}}_{\text{b}}^{\text{n}\prime }\nabla^{\text{b}}-\left(2\delta \omega_{\text{ie}}^{\text{n}}+\delta \omega_{\text{en}}^{\text{n}}\right)\\ \times \left({\hat{v}}^{\text{n}}-\delta {\text{v}}^{\text{n}}\right)-\left(2{\hat{\omega }}_{\text{ie}}^{\text{n}}+{\hat{\omega }}_{\text{en}}^{\text{n}}\right)\times \delta {\text{v}}^{\text{n}}+{\left({\text{C}}_{\text{n}}^{\text{n}\prime }\right)}^{\text{T}}{\text{C}}_{\text{b}}^{\text{n}\prime }{\text{w}}_{\text{a}}^{\text{b}}\\ {\dot{\varepsilon }}^{\text{b}}\text{=}0\\ {\dot{\nabla }}^{\text{b}}\text{=}0 \end{array}$$

### Range of Scaling Transformation

Through the INS error propagation model (18), the range of rotation transformation and translation transformation can be estimated. However, the range of scaling transformation cannot be directly calculated. To better describe the scaling transformation, the INS-indicated distance and the actual distance in a short period are expressed by the following equations:

(19) $$\begin{array}{r} d_{k-1,k}^{\text{INS}}=\frac{\left||{\text{v}}_{k-1}^{\text{INS}}+{\text{v}}_{k}^{\text{INS}}|\right|}{2}\cdot \Delta t \end{array}$$

(20) $$\begin{array}{r} d_{k-1,k}^{\text{real}}\approx \frac{\left||{\text{v}}_{k-1}^{\text{real}}+{\text{v}}_{k}^{\text{real}}|\right|}{2}\cdot \Delta t \end{array}$$

$d_{k\text{-}1,k}^{\text{INS}}$ and $d_{k-1,k}^{\text{real}}$ are, respectively, the INS-indicated distance and the actual distance between *k*-1th sampling point and *k*th sampling point. $v_{k}^{\text{INS}}$ and $v_{k}^{\text{real}}$ are the INS-indicated linear velocity and the actual linear velocity of *k*th sampling point. Δ*t* is the sampling period of the discrete time system.

Then, the relationship between $d_{k\text{-}1,k}^{\text{INS}}$and $d_{k-1,k}^{\text{real}}$ is derived:

(21) $$\begin{array}{r} \frac{d_{k-1,k}^{\text{real}}}{d_{k-1,k}^{\text{INS}}}=\frac{\left||{\text{v}}_{k-1}^{\text{real}}+{\text{v}}_{k}^{\text{real}}|\right|}{\left||v_{k-1}^{\text{INS}}+v_{k}^{\text{INS}}|\right|} \end{array}$$

Where ${\text{v}}_{k}^{\text{INS}}$ is described by an equation in the form:

(22) $$\begin{array}{r} v_{k}^{\text{INS}}=v_{k}^{\text{real}}+\delta v_{k} \end{array}$$

Here *δ****v***_*k*_ is the INS accumulated velocity error of kth sampling point. Accordingly, (21) is developed into the following form:

(23) $$\begin{array}{r} \frac{d_{k-1,k}^{\text{real}}}{d_{k-1,k}^{\text{INS}}}=\frac{\left||{\text{v}}_{k-1}^{\text{INS}}+{\text{v}}_{k}^{\text{INS}}-\delta {\text{v}}_{k\text{-}1}-\delta {\text{v}}_{k}|\right|}{\left||{\text{v}}_{k-1}^{\text{INS}}+{\text{v}}_{k}^{\text{INS}}|\right|} \end{array}$$

Denoting with *N* the number of sampling points on matching sequence, the constraint of scale transform has been derived:

(24) $$\begin{array}{r} S=\frac{\overset{N}{\underset{k=2}{\sum }}d_{k-1,k}^{\text{real}}}{\overset{N}{\underset{k=2}{\sum }}d_{k-1,k}^{\text{INS}}}=\frac{\overset{N}{\underset{k=2}{\sum }}||{\text{v}}_{k-1}^{\text{INS}}+{\text{v}}_{k}^{\text{INS}}-\delta {\text{v}}_{k\text{-}1}-\delta {\text{v}}_{k}||}{\overset{N}{\underset{k=2}{\sum }}||{\text{v}}_{k-1}^{\text{INS}}+{\text{v}}_{k}^{\text{INS}}||} \end{array}$$

## Affine Transformation Based ABC Algorithm

In order to achieve high positioning accuracy under large initial errors, this paper proposes an affine transformation based ABC algorithm. The proposed algorithm is specifically presented to deal with the gravity matching problem for underwater navigation system. The affine transformation based ABC algorithm introduces affine transformation to both initialization process and evolutionary process of ABC algorithm. In addition, the affine transformation satisfies the constraint conditions provided by the output characteristics of the INS.

### Objective Function and Initialization

The proposed algorithm use mean absolute difference (MAD) function as the objective function:

(25) $$\begin{array}{r} f\left(\text{x}\right)=\frac{1}{N}\overset{N}{\underset{k=1}{\sum }}|g_{k}^{\text{obs}}-g_{k}^{\text{x}}| \end{array}$$

Where ***x*** indicates a sequence of several consecutive position vectors. $g_{k}^{\text{obs}}$ is the measured gravity anomaly value of *k*th sampling point and $g_{k}^{\text{x}}$ is the corresponding gravity anomaly value on gravity anomaly base map of *k*th sampling point. *f*(***x***) is the objective function.

*fit*(***x***), the fitness value of ***x***, is calculated by (26):

(26) $$\begin{array}{r} fit\left(\text{x}\right)=\frac{1}{1+f\left(\text{x}\right)} \end{array}$$

Initialization process is the first step of the proposed algorithm. In ABC algorithm, the initial swarm composed of employed bees are given by (1). But the lower bound and the upper bound of the ***x*** in sequence matching are difficult to acquire. Thus, the initialization method should be redefined.

The proposed algorithm applies scaling transformation, rotation transformation, and translation transformation on the INS-indicated sequence to implement initialization. Translation transformation is implemented by choosing a random first element of initial swarm:

(27) $$\begin{array}{r} x_{{}_{i,1}}^{L}=x_{\min}^{L}+rand\left(0,1\right)\left(x_{\max}^{L}-x_{\min}^{L}\right) \end{array}$$

(28) $$\begin{array}{r} x_{{}_{i,1}}^{\lambda }=x_{\min}^{\lambda }+rand\left(0,1\right)\left(x_{\max}^{\lambda }-x_{\min}^{\lambda }\right) \end{array}$$

Where $x_{{}_{i,1}}^{L}$ and $x_{{}_{i,1}}^{\lambda }$ indicate the latitude and longitude of the first sampling point on sequence *i*, *i* = {1, 2, ⋯ , *SN*} and *SN* is the size of population. $x_{\max}^{L}$ and $x_{\min}^{L}$ are, respectively, the upper bound and the lower bound of latitude in search scope. $x_{\max}^{\lambda }$ and $x_{\min}^{\lambda }$ are, respectively, the upper bound and the lower bound of longitude in search scope.

Let $S_{i}^{\text{ini}}$ be the scale factor used for initialization sequence *i*. The value of $S_{i}^{\text{ini}}$ is calculated by (29):

(29) $$S_{i}^{\text{ini}}=\{\begin{array}{cc} rand(1,S), & S\geq 1\\ rand(S,1), & S<1 \end{array}$$

The expression of *S* is obtained from (24). The rotation angle used for initialization sequence *i* can be calculated by the following equation:

(30) $$\begin{array}{r} \beta_{i}^{\text{ini}}=rand\left(\beta_{\min}^{\text{ini}},\beta_{\max}^{\text{ini}}\right) \end{array}$$

Where $\beta_{\min}^{\text{ini}}$ and $\beta_{\max}^{\text{ini}}$ are the minimum angle error and the maximum angle error of a segment in INS-indicated trajectory. Thus, the rotation matrix used for initialization sequence *i* have been derived:

(31) $$\begin{array}{r} {\text{R}}_{i}^{\text{ini}}=\left[\begin{array}{cc} \cos\beta_{i}^{\text{ini}} & -\sin\beta_{i}^{\text{ini}}\\ \sin\beta_{i}^{\text{ini}} & \cos\beta_{i}^{\text{ini}} \end{array}\right] \end{array}$$

Let ${\text{X}}^{\text{INS}}=\left\{{\text{x}}_{1}^{\text{INS}},{\text{x}}_{2}^{\text{INS}},\ldots ,{\text{x}}_{N}^{\text{INS}}\right\}$ be the INS-indicated trajectory and ${\text{x}}_{i}^{\text{ini}}=\left\{{\text{x}}_{i,1}^{\text{ini}},{\text{x}}_{i,2}^{\text{ini}},\ldots ,{\text{x}}_{i,N}^{\text{ini}}\right\}$ be the *i*th initialization sequence. After above mentioned transformations have been performed, the initial swarm is obtained:

(32) $${\text{X}}_{I}^{\text{INS}\prime }=S_{i}^{\text{ini}}{\text{R}}_{i}^{\text{ini}}{\text{X}}^{\text{INS}}$$

(33) $${\text{T}}_{i}^{\text{ini}}={\text{x}}_{i,1}^{\text{ini}}-{\text{x}}_{1}^{\text{INS}\prime }$$

(34) $${\text{X}}_{i}^{\text{ini}}={\text{X}}_{i}^{INS\prime }\text{+}{\text{T}}_{i}^{\text{ini}}$$

Where ${\text{X}}_{i}^{\text{INS}\prime }$ is the intermediate sequence, ${\text{x}}_{i}^{\text{INS}\prime }$ is the first point in ${\text{X}}_{i}^{\text{INS}\prime }$. Additional translation vector need to be applied on generated trajectories if they are out of range. Let $init_{\min}^{L}$ and $init_{\max}^{L}$ be the maximum and minimum latitude of initial trajectory. $init_{\min}^{\lambda }$ and $init_{\max}^{\lambda }$ are the maximum and minimum longitude of initial trajectory. **T** = {*T*^*L*^, *T*^λ^}, the translation vector, can be expressed as:

(35) $$T^{L}=\{\begin{array}{ll} x_{\min}^{L}-init_{\min}^{L}, & init_{\min}^{L}<x_{\min}^{L}\\ x_{\max}^{L}-init_{\max}^{L}, & init_{\max}^{L}>x_{\max}^{L}\\ 0, & \text{other} \end{array}$$

(36) $$T^{\lambda }=\{\begin{array}{ll} x_{\min}^{\text{λ}}-init_{\min}^{\text{λ}}, & init_{\min}^{\text{λ}}<x_{\min}^{\text{λ}}\\ x_{\max}^{\text{λ}}-init_{\max}^{\text{λ}}, & init_{\max}^{\text{λ}}>x_{\max}^{\text{λ}}\\ 0, & \text{other} \end{array}$$

### Employed Bee Phase

With the initialization complete, an employed bee updates its food source in the neighborhood. Suppose ${\text{X}}_{i}^{\text{ini}}$ is the sequence to be updated, ${\text{X}}_{j}^{\text{ini}}=\left\{{\text{x}}_{j,1}^{\text{ini}},{\text{x}}_{j,2}^{\text{ini}},\ldots ,{\text{x}}_{j,N}^{\text{ini}}\right\}$ is selected randomly in the neighborhood of ${\text{x}}_{i}^{\text{ini}}$, *j* ∈ {1, 2, ⋯ , *i* − 1, *i* + 1, ⋯ , *SN*} and *SN* is the size of population. The evolution equation is derived through the relation between ${\text{x}}_{i}^{\text{ini}}$ and ${\text{x}}_{j}^{\text{ini}}$.

Denoting with ***Q*** the covariance matrix:

(37) $$\begin{array}{r} \text{Q}=\left[\begin{array}{cc} Q_{11} & Q_{12}\\ Q_{21} & Q_{22} \end{array}\right]=\overset{N}{\underset{k=1}{\sum }}\left({\text{x}}_{i,k}^{\text{ini}}-{\tilde{\text{x}}}_{i}^{\text{ini}}\right){\left({\text{x}}_{j,k}^{\text{ini}}-{\tilde{\text{x}}}_{j}^{\text{ini}}\right)}^{\text{T}} \end{array}$$

Where ${\tilde{\text{x}}}_{i}^{\text{ini}}$ and ${\tilde{\text{x}}}_{j}^{\text{ini}}$ are, respectively, the average value of all sampling points in ${\text{X}}_{i}^{\text{ini}}$ and ${\text{X}}_{j}^{\text{ini}}$. The eigenvalues of ***Q*** and the rotation angle from ${\text{X}}_{i}^{\text{ini}}$ to ${\text{X}}_{j}^{\text{ini}}$ are calculated through quaternion algorithm (Berthold, 1987):

(38) $$\begin{array}{rcl} \lambda_{1,2} & = & \pm {\left[{\left(Q_{11}+Q_{22}\right)}^{2}+{\left(Q_{21}-Q_{12}\right)}^{2}\right]}^{1/2}\\ \lambda_{3,4} & = & \pm {\left[{\left(Q_{11}-Q_{22}\right)}^{2}+{\left(Q_{21}+Q_{12}\right)}^{2}\right]}^{1/2} \end{array}$$

(39) $$\begin{array}{rcl} tg\left(\frac{\tau }{2}\right) & = & \left(Q_{11}+Q_{22}-\lambda_{\text{m}}\right)/\left(Q_{12}-Q_{21}\right) \end{array}$$

Where λ_μ_ (μ = {1, 2, 3, 4}) are four eigenvalues of ***Q***. λ_m_ is the maximum eigenvalue. τ is the rotation angle from ${\text{X}}_{i}^{\text{ini}}$ to ${\text{X}}_{j}^{\text{ini}}$. Then, the rotation angle used for ${\text{X}}_{i}^{\text{ini}}$ during employed bee phase is calculated by the following equations:

(40) $$\begin{array}{r} \beta_{i}^{\text{em}}=rand\left(-|\tau |,|\tau |\right) \end{array}$$

Notice that the angle between ${\text{X}}_{i}^{\text{ini}}$ and ***X***^INS^ after rotation should be in the range $\left(\beta_{\min}^{\text{ini}},\beta_{\max}^{\text{ini}}\right)$. Denoting with η be the rotation angle from ${\text{X}}_{i}^{\text{ini}}$ to ***X***^INS^, $\beta_{i}^{\text{em}}$ should meet the following in equation:

(41) $$\begin{array}{r} \beta_{\min}^{\text{ini}}+\eta <\beta_{i}^{\text{em}}<\beta_{\max}^{\text{ini}}+\eta \end{array}$$

Then, the following expression is obtained by (40) and (41):

(42) $$\begin{array}{r} \beta_{i}^{\text{em}}=rand\left(\max\left(-|\tau |,\beta_{\min}^{\text{ini}}+\eta \right),min\left(|\tau |,\beta_{\max}^{\text{ini}}+\eta \right)\right) \end{array}$$

Thus, the rotation matrix used for ${\text{X}}_{i}^{\text{ini}}$ during employed bee phase is calculated by (43):

(43) $$\begin{array}{r} {\text{R}}_{i}^{\text{em}}=\left[\begin{array}{cc} \cos\beta_{i}^{\text{em}} & -\sin\beta_{i}^{\text{em}}\\ \sin\beta_{i}^{\text{em}} & \cos\beta_{i}^{\text{em}} \end{array}\right] \end{array}$$

Let $L_{i}^{\text{ini}}$ and $L_{j}^{\text{ini}}$ be the length of ${\text{X}}_{i}^{\text{ini}}$ and ${\text{X}}_{j}^{\text{ini}}$. *L*^ins^ is the length of INS-indicated trajectory. The scale factor used for ${\text{X}}_{i}^{\text{ini}}$ during employed bee phase is calculated by the following equations:

(44) $$\begin{array}{r} S_{i}^{\text{em}}=rand\left(\min\left(\frac{L_{j}^{\text{ini}}}{L_{i}^{\text{ini}}},\frac{L_{i}^{\text{ini}}}{L_{j}^{\text{ini}}}\right),max\left(\frac{L_{j}^{\text{ini}}}{L_{i}^{\text{ini}}},\frac{L_{i}^{\text{ini}}}{L_{j}^{\text{ini}}}\right)\right) \end{array}$$

Notice that the scale factor between ${\text{X}}_{i}^{\text{ini}}$ and ***X***^INS^ after scaling transformation should meet the range on the right side of (29):

(45) $$\{\begin{array}{l} 1\;\cdot L^{\text{ins}}<L_{i}^{\text{ini}}\cdot S_{i}^{\text{em}}<S\;\cdot L^{\text{ins}},S\geq 1\\ S\cdot \;L^{\text{ins}}<L_{i}^{\text{ini}}\cdot S_{i}^{\text{em}}<1\;\cdot L^{\text{ins}},S<1 \end{array}$$

Denoting with $S_{\min}^{\text{em}}$ and $S_{\max}^{\text{em}}$ the lower bound and the upper bound of $S_{i}^{\text{em}}$. According to (44) and (45), $S_{i}^{\text{em}}$ is updated by (46):

(46) $$\begin{array}{rcl} S_{i}^{\text{em}} & = & rand\left(S_{\min}^{\text{em}},S_{m\text{ax}}^{\text{em}}\right) \end{array}$$

(47) $$\begin{array}{l} \begin{array}{ll} S_{\min}^{\text{em}}= & \{\begin{array}{l} \max(min(\frac{L_{j}^{\text{ini}}}{L_{i}^{\text{ini}}},\frac{L_{i}^{\text{ini}}}{L_{j}^{\text{ini}}}),\frac{L^{\text{ins}}}{L_{i}^{\text{ini}}}),\;S\geq 1\\ \max(min(\frac{L_{j}^{\text{ini}}}{L_{i}^{\text{ini}}},\frac{L_{i}^{\text{ini}}}{L_{j}^{\text{ini}}}),S\;\cdot \;\frac{L^{\text{ins}}}{L_{i}^{\text{ini}}}),\;S<1 \end{array} \end{array}\\ \begin{array}{ll} S_{\max}^{\text{em}}= & \{\begin{array}{l} \min(\max(\frac{L_{j}^{\text{ini}}}{L_{i}^{\text{ini}}},\frac{L_{i}^{\text{ini}}}{L_{j}^{\text{ini}}}),S\;\cdot \;\frac{L^{\text{ins}}}{L_{i}^{\text{ini}}}),\;S\geq 1\\ \min(\max(\frac{L_{j}^{\text{ini}}}{L_{i}^{\text{ini}}},\frac{L_{i}^{\text{ini}}}{L_{j}^{\text{ini}}}),\;\cdot \;\frac{L^{\text{ins}}}{L_{i}^{\text{ini}}}),\;S<1 \end{array} \end{array} \end{array}$$

${\text{X}}_{i}^{\text{ini}}$ is converted to ${\text{X}}_{i}^{\text{ini}\prime }$ after rotation transformation and scaling transformation have been performed:

(48) $$X_{i}^{\text{ini}\prime }=S_{i}^{\text{em}}R_{i}^{\text{em}}X_{i}^{\text{ini}}$$

Let ${\tilde{\text{x}}}_{i}^{\text{ini}\prime }$ be the average value of all sampling points in ${\text{X}}_{i}^{\text{ini}\prime }$ the translation vector is calculated by (49):

(49) $${\text{T}}_{i}^{\text{em}}=({\tilde{\text{x}}}_{i}^{\text{ini}}-{\tilde{\text{x}}}_{i}^{\text{ini}\prime })\cdot \;rand(-1,1)$$

Then, the employed bees update their sources according to the following equation:

(50) $${\text{X}}_{i}^{\text{em}}={\text{X}}_{i}^{\text{ini}\prime }+{\text{T}}_{i}^{\text{em}}$$

If the fitness value of ${\text{X}}_{i}^{\text{em}}$ is larger than the fitness value of ${\text{X}}_{i}^{\text{ini}}$, ${\text{X}}_{i}^{\text{ini}}$ will be replaced by ${\text{X}}_{i}^{\text{em}}$. At the end of the employed bee phase, all of current sequences are denoted by ***X***^em^.

### Onlooker Bee Phase and Scout Bee Phase

The probability of each sequence to be selected by the onlooker bee is calculated by (4). Suppose ${\text{X}}_{i}^{\text{em}}$ is the sequence selected by onlooker bee, the rotation matrix ${\text{R}}_{i}^{\text{on}}$, the scale factor $S_{i}^{\text{on}}$, and the translation vector ${\text{T}}_{i}^{\text{on}}$ used on ${\text{X}}_{i}^{\text{em}}$ during onlooker bee phase can be calculated by (43), (46), and (49). Thus, the onlooker bees update their sources according to the following equation:

(51) $$\begin{array}{r} {\text{X}}_{i}^{\text{on}}=S_{i}^{\text{on}}{\text{R}}_{i}^{\text{on}}{\text{X}}_{i}^{\text{em}}+{\text{T}}_{i}^{\text{on}} \end{array}$$

If the fitness value of ${\text{X}}_{i}^{\text{on}}$ is larger than the fitness value of${\text{X}}_{i}^{\text{em}}$, ${\text{X}}_{i}^{\text{em}}$ will be replaced by ${\text{X}}_{i}^{\text{on}}$. The best solution achieved so far is memorized. In addition, a sequence will be abandoned when limit, a control parameter, is exceeded for the source during scout bee phase. So it is replaced with randomly produced solution by (34). The pseudo of the proposal is given below.

**Table:** Procedure of the proposal

**Begin**
1. confirm search scope
2. initialize a matching sequence ${\text{X}}_{i}^{\text{ini}}$ at generation *t* = 0 with *trial*(*i*) = 0 using (34) for *i* = 1,2,…,*SN*
3. evaluate $f\left({\text{X}}_{i}^{\text{ini}}\right)$ for *i* = 1,2,…,*SN* and set the best solution as **X**_best_
4. **while** termination condition is not reached **do** //**employed bee phase**
1) set *k* = 1, **while** *k*<*SN*+1 **do**
Produce a new solution ${\text{X}}_{k}^{\text{em}}$ for ${\text{X}}_{k}^{\text{ini}}$ using (50)
if $f\left({\text{X}}_{k}^{\text{ini}}\right)>f\left({\text{X}}_{k}^{\text{em}}\right)$, ${\text{X}}_{k}^{\text{ini}}={\text{X}}_{k}^{\text{em}}$, trial(*i*) = 0
else *trial*(*i*) = *trial*(*i*)+1
end if *k* = *k*+1
**end while**
2) all of current sequences are denoted by **X**^em^
//**onlooker bee phase**
3) set *k* = 1, **while** *k*<*SN*+1 **do**
Select a sequence ${\text{X}}_{i}^{\text{em}}$ based on its probability of selection calculated using (4) and produce a new solution ${\text{X}}_{i}^{\text{on}}$ using (51)
If $f\left({\text{X}}_{i}^{\text{em}}\right)>f\left({\text{X}}_{i}^{\text{on}}\right)$, ${\text{X}}_{i}^{\text{em}}={\text{X}}_{i}^{\text{on}}$, *trial*(*i*) = 0
else *trial*(*i*) = *trial*(*i*)+1
end if
*k* = *k*+1
**end while**
4) all of current sequences are denoted by **X**^on^
//**scout bee phase**
5) denote the best solution in **X**^on^ as ${{\text{X}}_{\text{best}}}^{\prime }$
if $f\left({\text{X}}_{\text{best}}\right)>f\left({{\text{X}}_{\text{best}}}^{\prime }\right)$, ${\text{X}}_{\text{best}}={{\text{X}}_{\text{best}}}^{\prime }$
end if
6) reinitialize *i*th sequence if *trial*(*i*) > *limit*
7) *t* = *t*+1
8) all of current sequences are denoted by **X**^ini^
**end while**
5. output **X**_best_
**End**

## Simulation and Analysis

### Simulation Parameters

**Figure 1:**
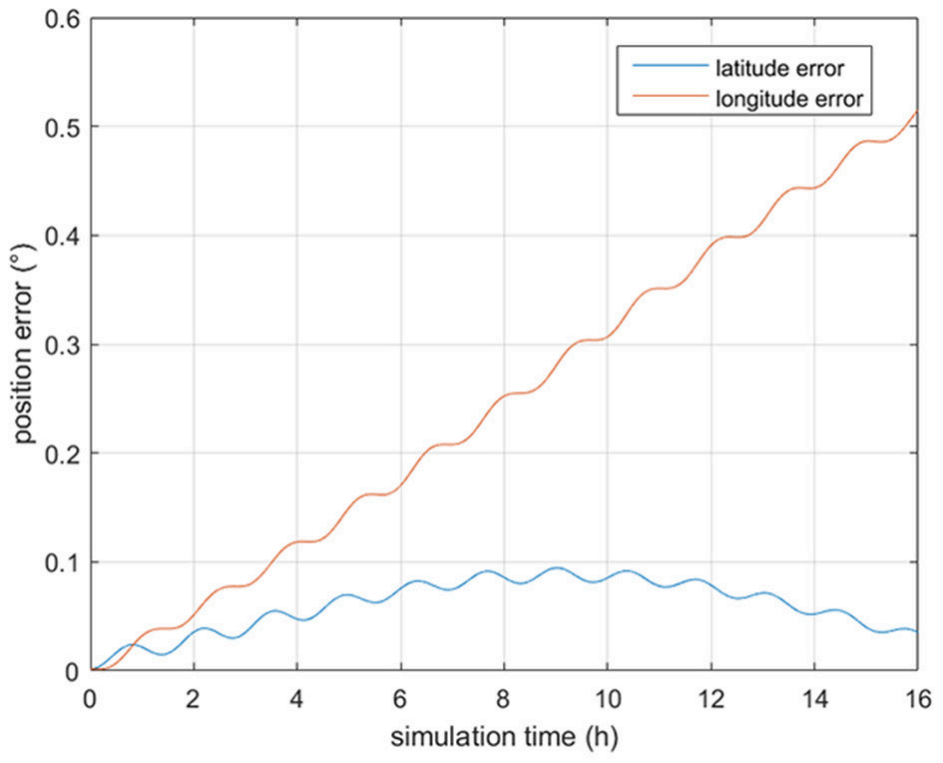
The INS position error.

In order to test the feasibility of our approach, numerous simulation experiments have been performed. An INS indicated trajectory start from (156°E, 20°N) is generated and the INS relevant parameters are listed in Table 1. The INS position error is shown in Figure 1 (Supplementary Data Sheet 1). As seen from Figure 1, the position error of the generated trajectory exhibits a Scuhler oscillation of 84.4 min.

**Table 1:** Simulation condition of INS.

**Parameters**	**Quantity**	**Unit**
Gyro constant drift	0.02	°/h
Gyro random drift (1σ)	0.02	°/h
Accelerometer constant bias	100	μg
Accelerometer random bias (1σ)	100	μg
Velocity	7.71	m/s
Acceleration	0	m/s
Initial angle error	0	°
Azimuth angle	60	°
Initial longitude error	0.1	′
Initial latitude error	0.1	′
Simulation time	16	h

**Figure 2:**
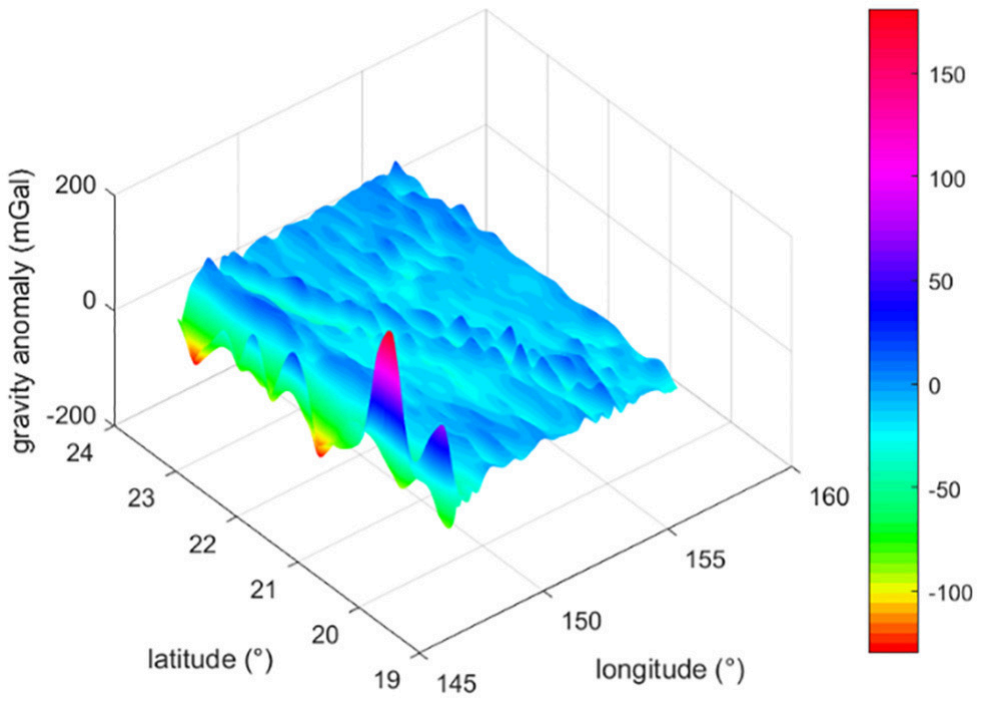
3-D gravity anomaly base map.

In order to evaluate the algorithm presented in this work, a gravity anomaly base map is required. In the simulations tests, goco05c model (Fecher et al., 2017) is used to calculate the gravity anomaly in the area from (147.6°E, 19.5°N) to (156.6°E, 24°N). After interpolated, the grid step is converted to 0.3′. The 3-D map of the gravity anomaly data is shown in Figure 2 (Supplementary Data Sheet 1). The gravity anomaly relevant parameters are shown in Table 2.

**Table 2:** Parameters of gravity anomaly base map.

**Model name**	**Goco05c**
Number of grid points	900 × 1800
Grid step	0.3′
Minimum value	−130. 039 mGal
Maximum value	180.902 mGal
Mean	−15.246 mGal

**Figure 3:**
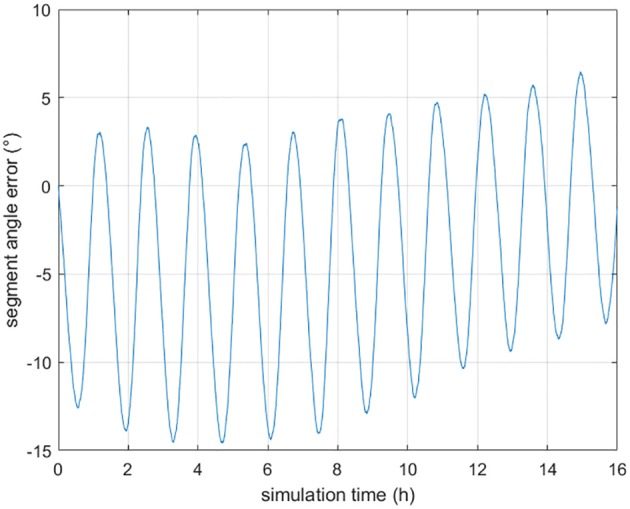
The INS-indicated segment angle error.

Figure 3 shows the angle error of a segment in INS-indicated trajectory. It is obtained that the angle error is vary in a small range and the maximum segment angle error is 15°. Thus, $\beta_{\min}^{\text{ini}}$ and $\beta_{m\text{ax}}^{\text{ini}}$ are set as −15°and 15°, respectively. The proposed algorithm uses the 3σ principle to confirm the search scope (Han et al., 2016). Table 3 shows the configuration of the proposed algorithm. In this paper, optimal parameter values of affine transformation based ABC are obtained based on experience.

**Table 3:** Configuration of the proposed algorithm.

**Maximum iterations**	**500**
Limit	20
Population size	10
Number of sampling points per sequence	12
Sampling interval	5 min
The variance of gravity anomaly measurement noise	1 mGal

### Comparison Between Basic ABC and Affine Transformation Based ABC

**Figure 4:**
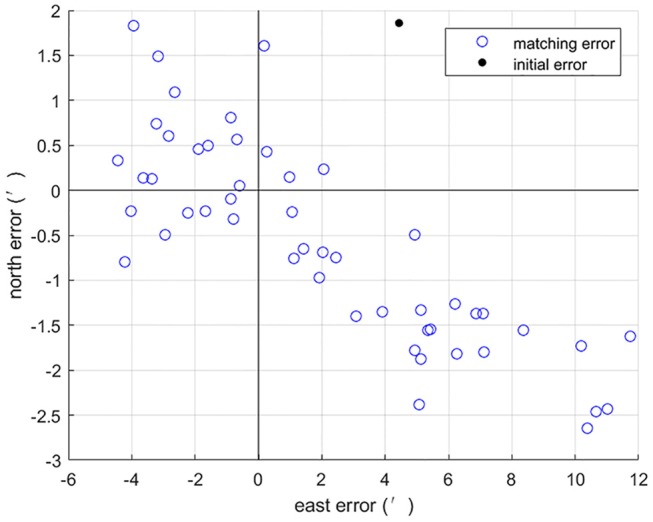
Position errors of basic ABC.

**Figure 5:**
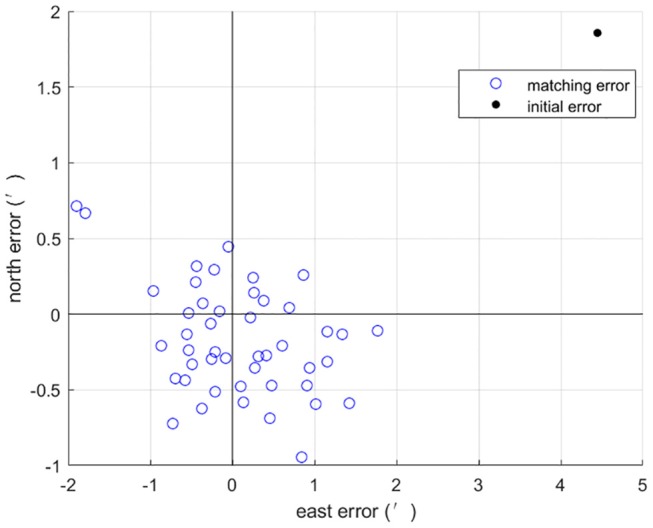
Position errors of affine transformation based ABC.

Based on the parameters above, the first set of 50 Mont Carlo simulation tests were done on the trajectory after 3 h of sailing. Simulation results of introducing sequence matching strategy into ABC algorithm has better positioning precision and matching probability than the basic ABC, as shown in Figures 4, 5. Statistical results of simulation tests are given in Table 4. In Table 4, a matching result is considered to be a successful matching if the position error is within 2′.

**Table 4:** Comparison of 50 Mont Carlo simulation results between basic ABC and affine transformation based ABC.

**Algorithm**	**Initial error (**′**)**	**Mean error (**′**)**	**Matching probability (%)**
Basic ABC	4.82	4.22	28
Affine transformation based ABC	4.82	0.73	96

### Comparison Between ICCP, Improved-ABC and Affine Transformation Based ABC

Due to ICCP is a widely used gravity matching algorithm and improved-ABC algorithm (Gao et al., 2014) is presented to tackle the similar problem. We use these algorithms as references, comparing the results of ICCP algorithm and improved-ABC algorithm with the proposed algorithm.

**Figure 6:**
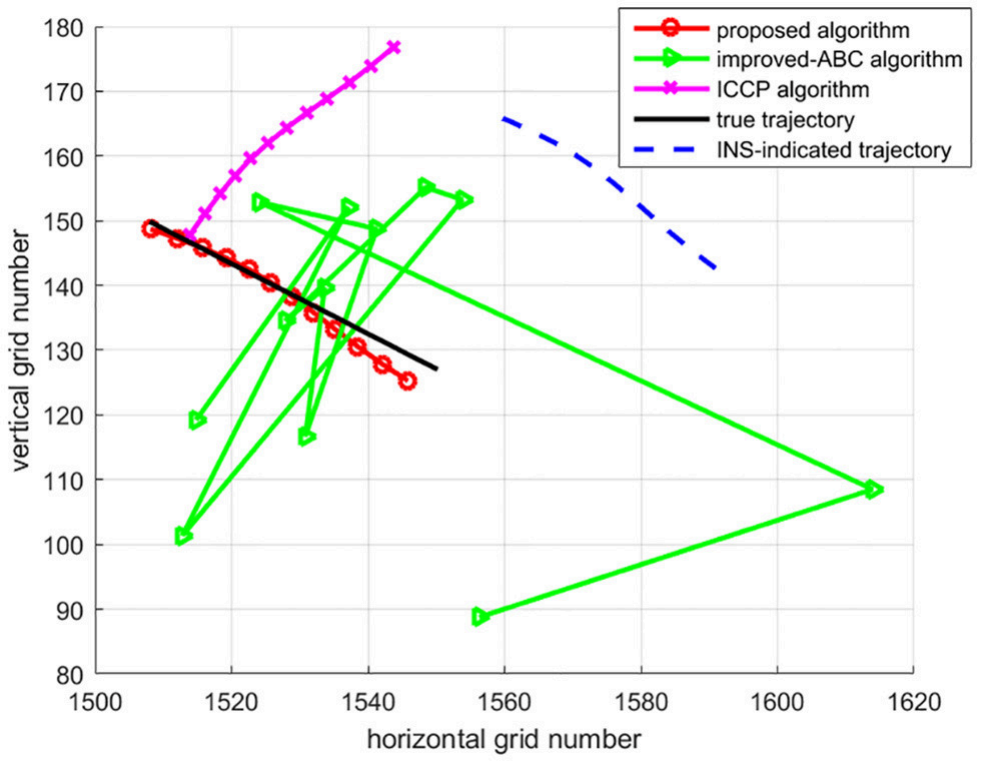
Matched trajectory.

**Figure 7:**
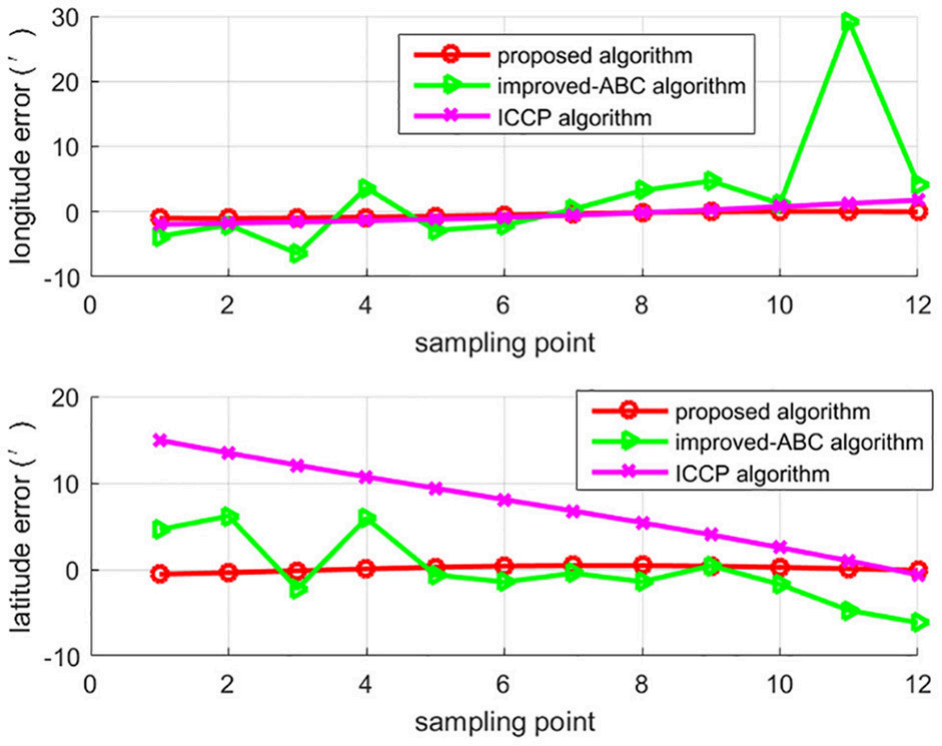
The average error of longitude and latitude.

To test algorithms under large initial position errors, the second set of 50 Mont Carlo simulation tests were done on the trajectory after 7 h of sailing using the above three algorithms. Trajectories of matching results are shown in Figure 6, each trajectory is a typical one in 50 Mont Carlo simulations. Figure 7 indicates the average error of longitude and latitude of each sampling point in 50 Mont Carlo simulations. In order to better evaluate the proposed algorithm, the statistical results of all the three algorithms are given in Table 5.

**Table 5:** Statistical results of the matching errors.

**Algorithm**	**Initial position error (km)**	**Mean error (km)**	**Standard deviation (km)**
ICCP	22.47	25.85	0
Improved-ABC	22.47	15.95	8.70
Proposed algorithm	22.47	2.05	0.55

Simulation results in Figures 6, 7, and Table 5 show that the matching accuracy of the proposed algorithm is significantly superior to the other two algorithms under large initial position errors.

**Figure 8:**
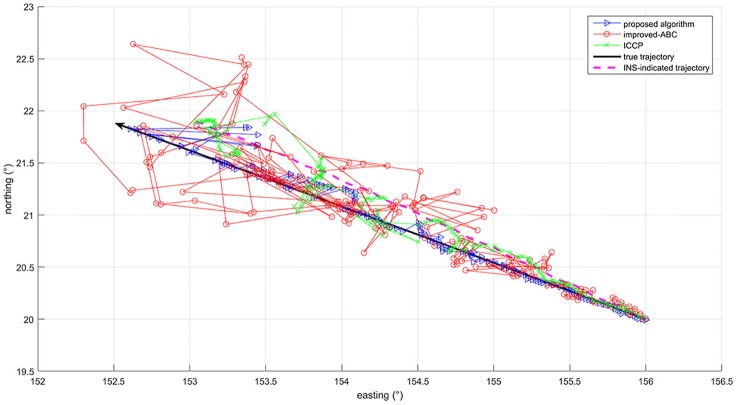
Matching results of the whole trajectory.

To better observe the effect of initial errors on these three algorithms, matching results of the whole trajectory within 16 h are shown in Figure 8 and Table 6. The results are divided into three time periods for statistic. In Table 6, a matching result is considered to be a successful matching if the position error is within 5′. It can be concluded that the convergence speed of ICCP algorithm decreases a lot with the increase of initial position errors. While the convergence speed of improved-ABC algorithm is not affected by initial position errors. The proposed algorithm converge slower than improved-ABC algorithm and much faster than ICCP algorithm under large initial errors. In Figure 8, at the beginning, all of the three algorithms can hold high positioning accuracy. But with the initial errors and the search scope increase, the accuracy of ICCP algorithm and improved-ABC algorithm decrease significantly, whereas the proposed algorithm can guarantee much higher matching accuracy.

**Table 6:** Statistical results within 16 h.

**Time period**	**Algorithm**	**Average matching time (s)**	**Mean error (**′**)**	**Matching probability (%)**
0–5 h	Improved-ABC	0.05	5.96	57
	ICCP	0.67	3.71	67
	Proposed algorithm	0.21	1.00	100
5–10 h	Improved-ABC	0.04	14.71	20
	ICCP	3.41	9.22	30
	Proposed algorithm	0.82	3.23	82
10–16 h	Improved-ABC	0.05	27.47	9
	ICCP	9.88	22.33	0
	Proposed algorithm	1.75	6.63	73

## Conclusion

Gravity aided inertial navigation system can solve the problem of INS error accumulation and ensure the concealment of INS. However, there are still many problems in the field of gravity aided navigation system. In this paper, we focus on the problem of how to ensure the matching accuracy under the large initial errors condition. In order to avoid mismatching under large initial errors, an affine transformation based ABC algorithm is adopted. The simulation results show that the proposed algorithm can achieve high matching precision under the condition of large initial errors.

In addition to ABC algorithm, other evolutionary algorithms can also be used to solve the gravity matching problem. How to introduce the sequence matching strategy in other evolutionary algorithms? How are the effects of these algorithms after introducing the sequence matching strategy? These need to be further studied in the future. What's more, the matching accuracy of gravity matching algorithm is highly correlated with the precision and resolution of the gravity anomaly base map. This paper focuses on the matching algorithm for large initial errors. How to perform a gravity matching algorithm in the area with low uniqueness should be discussed in the next step.

## Author Contributions

TD and LM designed the study. TD, LM, and HS performed the simulations. All of the authors analyzed the data. All authors contributed to the writing and editing of the manuscript.

### Conflict of Interest Statement

The authors declare that the research was conducted in the absence of any commercial or financial relationships that could be construed as a potential conflict of interest.

## Supplementary Material

Supplementary Data Sheet 1 Gravity base map and the INS data.
